# Body landmarks and genetic algorithm-based approach for non-contact detection of head forward posture among Chinese adolescents: revitalizing machine learning in medicine

**DOI:** 10.1186/s12911-023-02285-2

**Published:** 2023-09-11

**Authors:** Guang Yang, Shichun He, Deyu Meng, Meiqi Wei, Jianwei Cao, Hongzhi Guo, He Ren, Ziheng Wang

**Affiliations:** 1 Chinese Center of Exercise Epidemiology, Northeast Normal University, Renmin Street, Changchun, 130024 Jilin China; 2 AI Group, Intelligent Lancet LLC, 2108 N Street, Sacramento, 95816 CA USA; 3 Graduate School of Human Sciences, Waseda University, Tokorozawa, 169-8050 Saitama Prefecture Japan; 4 Advanced Research Center for Human Sciences, Waseda University, Tokorozawa, 169-8050 Saitama Prefecture Japan

**Keywords:** Forward head posture, Non-contact detection, Feature engineering, Machine learning

## Abstract

Addressing the current complexities, costs, and adherence issues in the detection of forward head posture (FHP), our study conducted an exhaustive epidemiologic investigation, incorporating a comprehensive posture screening process for each participant in China. This research introduces an avant-garde, machine learning-based non-contact method for the accurate discernment of FHP. Our approach elevates detection accuracy by leveraging body landmarks identified from human images, followed by the application of a genetic algorithm for precise feature identification and posture estimation. Observational data corroborates the superior efficacy of the Extra Tree Classifier technique in FHP detection, attaining an accuracy of 82.4%, a specificity of 85.5%, and a positive predictive value of 90.2%. Our model affords a rapid, effective solution for FHP identification, spotlighting the transformative potential of the convergence of feature point recognition and genetic algorithms in non-contact posture detection. The expansive potential and paramount importance of these applications in this niche field are therefore underscored.

## Introduction

Forward Head Posture (FHP) is a pervasive phenomenon, generally characterized as an anterior cervical posture [1], where the cranium juts forward of the sagittal plane and appears to be positioned anterior to the body’s central alignment [2–7]. This abnormal carriage triggers muscular tension and shortening [8], which impedes the range of motion in the cervical spine, fostering the emergence of muscle imbalances, discomfort, and fatigue [9]. Furthermore, as the upper cervical spine extends, alterations also manifest within the lumbar spine, impacting the postural alignment of the entire upper torso [10]. The potential repercussions of these changes are manifold and irreversible. For instance, poor posture is a potent predictor of musculoskeletal disorders, especially as we age [1]. These adaptations could diminish the diaphragm’s mobility and strength, disrupt typical respiratory mechanics and fundamentally transform the respiratory mechanism [11–16], even more serious outcomes, like dysphagia [17, 18] and lifelong disability [5, 19]. Unfortunately, the ubiquity of electronic screen usage in learning and work environments has led to a surge in FHP cases, exhibiting a positive correlation with age [20]. Alarmingly, FHP onset often occurs in childhood, with a reported prevalence rate of up to 63% among adolescents aged 12-16 [10]. Therefore, early recognition of the occurrence of FHP among adolescents is crucial to provide targeted treatment and intervention as early as possible.

Traditional clinical diagnosis predominantly relies on digital head posture measurement systems to verify the presence of FHP [19]. More researchers are trying to develop head posture measurement devices and technologies such as Head-mounted motion trackers, Nintendo Wiimotes (Nintendo Co., Kyoto, Japan) [21] and Microsoft’s Face-Tracking Software Development Kit [22], which have been utilized to implement head pose measurements. However, these tools, which range from head-mounted motion trackers to advanced software development kits, remain prohibitively costly for wide-scale clinical applications, are often inaccessible in many regions, or may be unsuitable for non-compliant patient groups.

In the wake of rapid advancements in deep learning, the potential to automatically detect and filter task-relevant features without direct human expert intervention presents more objective and cost-effective alternatives [23]. Several models, including Hope Net [24] and OpenPose [25], have demonstrated proficiency in head pose detection. Nonetheless, there remains a notable absence of models dedicated to FHP classification, which could aid clinicians in reducing workload and enhancing diagnostic accuracy. Consequently, the central question arises: how can we exploit these models more effectively for FHP prediction?

Accordingly, this study collated FHP data from the Chinese adolescent population, and developed an end-to-end early prediction model for FHP. In this investigation, we aim to explore potential methodologies and strategies for maximizing the utility of deep learning models in FHP prediction. Our goal is not only to contribute to the body of knowledge in this specific domain, but also to provide practical tools and recommendations that can be directly implemented in clinical settings. By addressing this central question, we hope to facilitate more accurate and efficient FHP detection, promoting better patient outcomes and advancing the field of posture correction.

## Materials and methods

This research, approved by the Northeast Normal University Ethics Committee (NC2020112102), used photogrammetry for FHP diagnosis, employing the ’ex-body’ apparatus and manual image annotation. Key features relating to FHP were identified using OpenPose, and genetic algorithms optimized feature selection. The data informed the training of three machine learning and deep learning models, considering sample imbalance and implementing cross-validation. Performance metrics, assessed with the Clopper-Pearson method, provided statistical analysis of the model’s accuracy.

### Participants

In collaboration with both primary and secondary educational institutions within Changchun, China, four distinct schools generously consented to partake in our investigative study. These included the Dong’an Experimental Middle School and Dong’an Experimental Primary School of Northeast Normal University, along with Changchun Nanhu Experimental Middle School and Yangpu Middle School.

We instituted specific criteria to delineate the subjects appropriate for inclusion within our research scope. The inclusion parameters demanded that the subjects be within the age bracket of 10 and 19 years, without any communicative impairments. However, after collecting the data, we found that the maximum age of the subjects included in this study was 15 years and the minimum age was ten years. Inversely, our exclusion guidelines determined the omission of any subject suffering from significant physical illnesses, congenital anomalies, or skeletal-related ailments. Subjects who had experienced hospitalization or had undergone any form of medical treatment within the prior three-month period were also systematically excluded from the study.

### Data collection

We utilized a photogrammetric methodology characterized by substantial inter-rater reliability, to determine the incidence of FHP within the study sample [26]. The postural evaluation was facilitated using an apparatus known as ’ex-body’ (Lantian Medical Equipment Co. Ltd., Beijing, China), engineered for diagnosing subjects presenting FHP. This system comprises a grid-integrated monitor, imaging apparatus, and a computer system designed to mark anatomical landmarks. It enables the evaluator to denote bodily landmarks of the subjects meticulously, enhancing the precision of the assessment.

The assessment environment necessitated a dimly lit room, with an approximate distance of 3 meters separating the camera from the screen. The evaluator was equipped with a computer terminal to administer the imaging procedure, with the resultant data stored as image files within the computer system. The data acquisition was carried out entirely via online means, with subsequent image data analyses conducted offline. Subject preparation required attire consisting of snug-fitting shorts and short-sleeved tops, and a standing position with the sagittal plane orthogonal to the screen. They were instructed to stand barefoot, placing their left shoulder adjacent to the screen at a distance of 10 cm, adopting a relaxed stance with arms folded across the abdomen. Upon subject alignment with the prescribed position, researchers operated the system to capture the necessary images, with each assessment session enduring approximately 30 seconds.

For image analysis, both standard head postures and occurrences of FHP were considered. Three expert postural assessors were employed to manually annotate the images. The definitive annotation adhered to the majority rule, and this consensus formed the basis for the final labeling.

### Body landmarks recognition

The dataset procured for this investigation consisted of images that necessitated additional refinement processes. To facilitate this, the open-source initiative OpenPose [25] was utilized. Recognized for its exemplary in the realm of human pose identification, this utility is undergirded by “Two-Branch Multi-Stage Convolutional Neural Network”, effectively bifurcates the algorithm into two distinctive branches. The first is harnessed to generate Confidence Maps (S), whilst the second is engaged to produce Part Affinity Fields (L). The former corresponds to heatmaps, while the latter aligns with vectormaps. Here, $\text {S} = (\text {S}_{1}, S_{2}, \ldots , \text {S}_{\text {j}})$ is indicative of the heatmap, where ’j’ represents the count of skeletal points targeted for detection. In parallel, $\text {L} = (\text {L}_{1}, \text {L}_{2}, \ldots , \text {L}_{\text {C}})$ symbolizes the vector map, where ’C’ epitomizes the logarithm of the joint that is subject to examination.

**Fig. 1:**
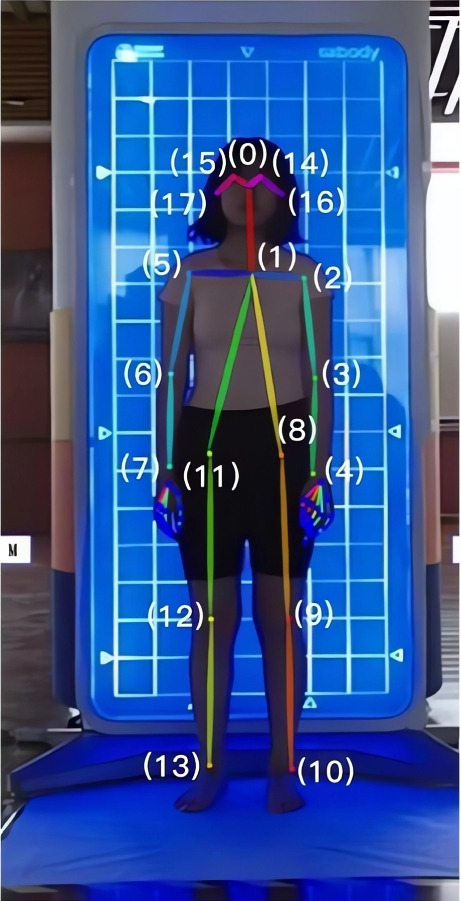
Schematic diagram of body landmarks after recognized with OpenPose (Frontal View)

OpenPose performs commendably in both singular and plural 2-D pose estimation contexts, capabilities encompass the detection of thirteen critical landmarks integral to the human skeletal structure: the nose (0), neck (1), right shoulder (2), right elbow (3), right wrist (4), left shoulder (5), left elbow (6), left wrist (7), right hip (8), right knee (9), right ankle (10), left hip (11), left knee (12), left ankle (13), right eye (14), left eye (15), right ear (16), and left ear (17). To optimize the visual clarity of each detected bodily landmark following their recognition, a frontal viewpoint of the subject was adopted. This is depicted through a representative image in Fig. 1.

### Feature extraction

The prominent characteristic of FHP is a diminished anterior cervical tilt consequent to the forward motion of the head. Given that OpenPose operates as a 2D human pose estimation algorithm, we observed that the left shoulder, neck, and right shoulder tend to overlap when OpenPose is employed for body landmarks recognization in a subject’s side view. Therefore, in light of the attributes of FHP patients and the completion of all assessments via side view, we extracted multiple angles akin to the cranial vertebral angle utilizing the subject’s right shoulder as the vertex to construct the features. Finally, our study designates four anatomical points: the Right Acromion Point (RAP), the highest point of the shoulder at the outer edge, as marked by the acromion process of the scapula (coordinates x0, y0); the Nasal Point (NP) (coordinates x1, y1); the Right Pupil Centre Point (RPCP), representing the eye’s center (coordinates x2, y2); and the Right External Auditory Meatus Point (REAMP) (coordinates x3, y3).

Subsequent to their identification, these points facilitate the computation of multiple angles, indicative of the forward extension of the head, with the RAP serving as the apex:

1. Nasal-Acromion-Horizontal Angle (NAH). This angle is formed at the intersection of a line connecting the NP and the RAP and a horizontal line passing through the RAP.
2. Ear-Nasal-Acromion Angle (ENA). The angle between the line connecting the REAMP and the RAP and the line connecting the NP and the RAP is defined as the ENA.
3. Pupil-Acromion-Horizontal Angle (PAH). Similar to the NAH, the PAH is the angle between the line connecting the RPCP and the RAP and a horizontal line through the RAP.
4. Ear-Acromion-Horizontal Angle (EAH). The EAH is defined as the angle formed between the line connecting the REAMP and the RAP and the horizontal line passing through the RAP.

Each angle was determined utilizing the cosine theorem, where A denotes the angle of the triangle and a, b, and c represent its sides. The resultant cosine formula for each angle can be depicted as in Eq. (1):

1 $$\begin{aligned} \cos (A) =\frac{b^{2}+c^{2}-a^{2}}{2 b c}, \end{aligned}$$

**Fig. 2:**
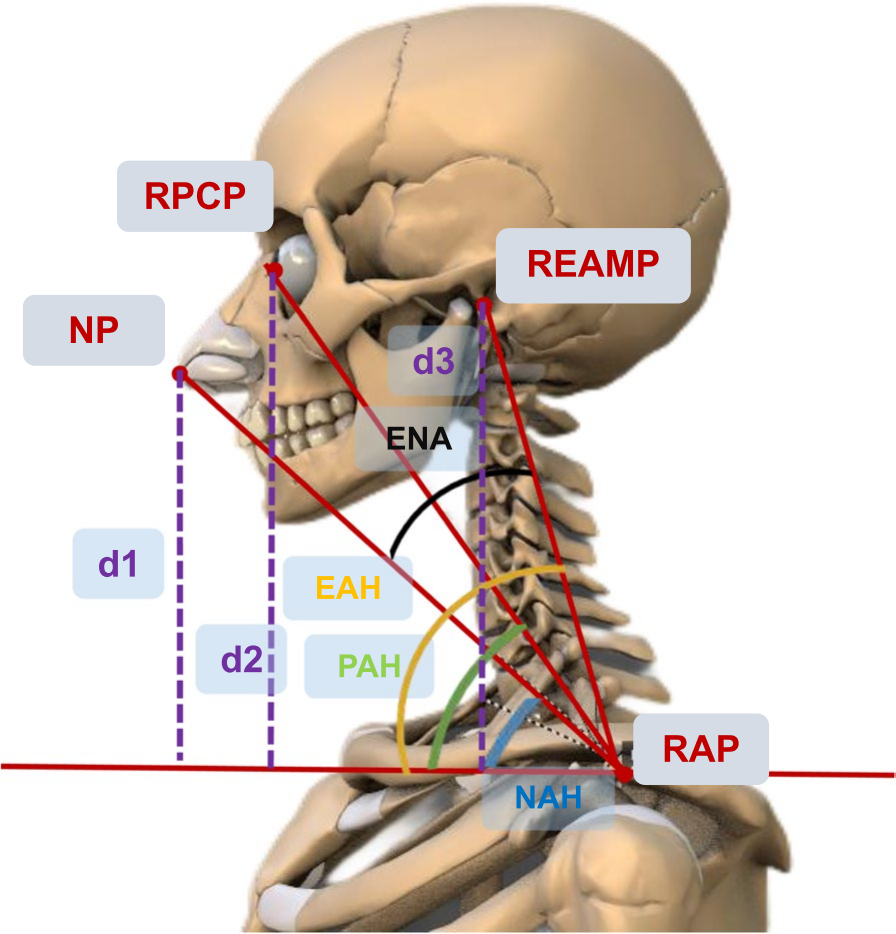
Process of feature extraction based on OpenPose. NAH: Nasal-Acromion-Horizontal Angle; ENA: Ear-Nasal-Acromion Angle; PAH: Pupil-Acromion-Horizontal Angle; EAH: Ear-Acromion-Horizontal Angle

Thus, the specific values of NAH, ENA, PAH, and EAH, which are of primary interest to our study, can be discerned from their corresponding cosine values. In addition, recognizing that individual variations in ear, nose, and eye positions can potentially influence the four aforementioned angles, we also computed vertical distances from the NP to the RAP (d1), from the RPCP to the RAP (d2), and from the REAMP to the RAP (d3). Figure 2 presents a visual illustration of the feature extraction process, with body landmarks identified employing OpenPose for accurate landmark detection.

### Feature selection based on genetic algorithm

Feature selection represents a critical step in model training, which is designed to refine the input feature set. This process aims to decrease model complexity, augment generalization, mitigate overfitting risk, and boost model efficiency. By focusing on a narrower range of features, the model is better equipped to manage unfamiliar data by suppressing noise and unnecessary information.

The genetic algorithm, an optimization technique inspired by natural selection and genetic processes, presents an innovative solution to intricate problems through the simulation of biological evolution [27]. The genetic algorithm generates a population from a potential feature subset, utilizing the fitness function *f*(*x*) to guarantee the chosen feature subset’s high fitness value, thereby maximizing their influence on model performance. The optimal feature subset, as determined by this process, aims to contain the minimum number of features to decrease recognition costs and enhance the speed of model inference. The formula for the fitness function is as Eq 2:

2 $$\begin{aligned} f\left( X_{i}\right) =f\left( x_{1}, x_{2}, \ldots , x_{k}\right) =C \frac{\frac{\sum _{i=1}^{k} N\left( x_{i}\right) }{P}+(\log _{n}-\log _{m})}{\sum _{i=1}^{k} x_{i}}, \end{aligned}$$

where, $X_{i}$ denotes the subset of possible features; $x_{i}$ denotes the feature term, which is recorded as 1 if included in the feature subset and 0 if excluded; *k* denotes the number of features in the feature subset; *C* denotes the scale factor, which is utilized to limit the fitness; *P* denotes the total number of features; $N(x_{i})$ denotes the number of times a feature is repeated in the solution process; $\log _{n}-\log _{m}$ denotes the contribution of the current feature subset to the model performance, *n* is the total number of a category in the labels, *m* is the total number of correct predictions of a label in the training set; $\sum _{i=1}^{k} x_{i}$ is the number of features in the current feature subset. In this study, we leverage genetic algorithms to derive a subset of pertinent features, incorporating basic subject characteristics (gender, age, height, weight, and BMI) as well as anatomical features extracted based on OpenPose (NAH, ENA, PAH, EAH, d1, d2, and d3). Specifically, at first, we took the data containing all the features and employed the ’Pycaret’ library in python to select the top three models in terms of performance. Then, we employed the data containing all the features to select features for the three models in sequence using genetic algorithm. Through the above process, we determined the best combination of features and the best model.

### Classification with classic models

To construct the dataset, we employed features ascertained through a robust process of feature selection and evaluation, with the ultimate goal of identifying an optimally performant machine learning model capable of FHP detection with high precision. Due to the issue of sample imbalance, we balanced the dataset and employed a ten-fold cross-validation strategy. The training data was subsequently fed into three distinct machine learning models: Extra Tree Classifier (ETC), Gradient Boosting Classifier (GBC), and XGBoost Classifier (XGB). The models’ performance was then assessed using a separate testing dataset.

To evaluate the diagnostic efficacy of the models, we computed a suite of performance metrics including accuracy, sensitivity, specificity, positive predictive value, and negative predictive value based on the prediction results.

The experiments were carried out by means of the PyCharm development tool. The employed computational hardware consisted of an Intel(R) Core i9 central processing unit, a NVIDIA GeForce RTX 3080 GPU, and 10 GB of random-access memory.

### Statistical analyses

The uncertainty inherent in statistical estimates can be quantified using confidence intervals, providing a valuable tool for more dependable statistical inferences and decision-making processes by researchers. The Clopper-Pearson method, founded on the binomial distribution’s cumulative distribution function, considers the probability mass at the confidence interval’s extremities and thus, can provide an exact confidence interval. Hence, we utilized the Clopper-Pearson approach to compute 95% confidence intervals for the metrics of sensitivity, accuracy, specificity, positive predictive value, and negative predictive value. These calculations were performed using python 3.8.

## Results

### Subjects

**Fig. 3:**
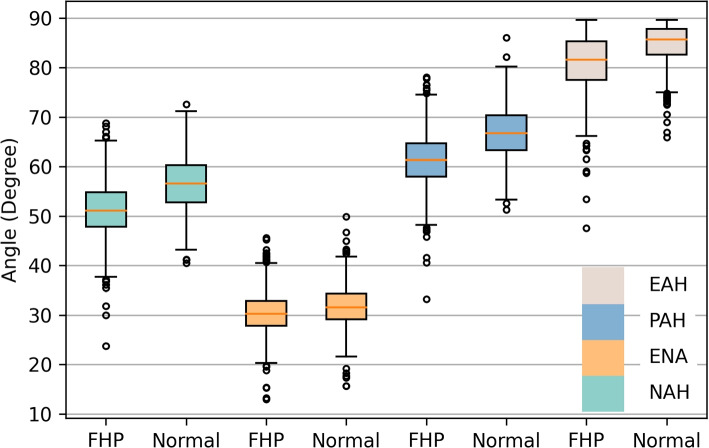
Comparison of each angle of FHP subjects and non-FHP subjects. The FHP patients showed a smaller angle compared with the normal. NAH: Nasal-Acromion-Horizontal Angle; ENA: Ear-Nasal-Acromion Angle; PAH: Pupil-Acromion-Horizontal Angle; EAH: Ear-Acromion-Horizontal Angle

Table 1 presents the demographic variables of the participants. A total of 1891 participants were initially recruited, from which 1651 samples were gathered after excluding data of inferior quality due to factors such as inadequate lighting and non-standard standing postures. The resultant dataset comprised 627 normal samples and 1024 abnormal samples. Given the presence of sample imbalance, we employed oversampling to balance the data. For model training, we allocated 90% of the samples, reserving the remaining 10% as a test set to evaluate the model’s performance. This test set encompassed 165 adolescents, among which 103 (62.4%) were diagnosed with FHP. As depicted in Fig. 3, the features extracted were consistent with the head and neck features observed in FHP patients.

**Table 1:** Demographic characteristics of subjects

Items	FHP ^a^ (n = 1024)	Normal (n = 627)
Gender (Female/Male)	542/482	331/296
singleton (Yes/No)	773/251	296/332
Age (years old)	12.08 ± 1.15	12.09 ± 1.17
Height (cm)	160.38 ± 9.22	158.18 ± 11.09
Mass (kg)	55.48 ± 16.16	54.18 ± 15.43
Muscle Mass (kg)	39.68 ± 8.96	38.58 ± 8.54
Fat-Free Mass (kg)	42.49 ± 9.37	41.31 ± 8.95
Skeletal Muscle Mass (kg)	23.81 ± 5.38	23.15 ± 5.12

^a^ FHP denotes forward head posture

### Comparison of models

Body landmarks and genetic algorithm-based data were subjected to three separate machine learning models while emphasizing the pivotal role of body key point recognition methods in our investigation. Concurrently, three distinct deep-learning models were trained using raw image data and their corresponding labels. Detailed performance indicators of each model using the test set are delineated in Table 2. Our findings designate the Extra Tree Classifier algorithm as the leading performer across multiple metrics. In the context of FHP prediction, the Extra Tree Classifier model achieved an accuracy of 82.4% (95% CI, 75.7% - 87.9%), sensitivity of 80.6% (95% CI, 71.6% - 87.7%), specificity of 85.5% (95% CI, 74.2% - 93.1%), positive predictive value of 90.2% (95% CI, 82.2% - 95.4%) and negative predictive value of 72.6% (95% CI, 60.9% - 82.4%).

**Table 2:** Results of performance comparison of classification modules

Modules	Accuracy (%, 95% CI)	Sensitivity (%, 95% CI)	Specificity (%, 95% CI)	PPV^a^ (%, 95% CI)	NPV^b^ (%, 95% CI)
XGBoost	78.2 (71.1, 84.2)	81.6 (72.7, 88.5)	72.6 (59.8, 83.1)	83.2 (74.4, 89.9)	70.3 (57.6, 81.1)
GBC ^c^	81.2 (74.4, 86.9)	78.6 (69.5, 86.1)	85.4 (74.2, 93.1)	90.0 (81.9, 95.3)	70.7 (59.0, 80.6)
ETC ^d^	82.4 (75.7, 87.9)	80.6 (71.6, 87.7)	85.5 (74.2, 93.1)	90.2 (82.2, 95.4)	72.6 (60.9, 82.4)

^a^ PPV denotes positive predictive value; ^b^ NPV denotes negative predictive value; ^c^ Gradient Boosting Classifier; ^d^ Extra Tree Classifier

### Results of ablation study

To ascertain the robustness of the selected features and to identify the most optimal feature combination for superior model performance, we conducted an ablation study on the top-performing model, the Extra Tree Classifier, employing the following four features: Neck Axis Height (NAH), Ear-Neck Angle (ENA), Posterior Axis Height (PAH), and Ear-Axis Height (EAH). The optimal combination of features, comprising “NAH, ENA, PAH, and EAH”, was selected as the benchmark for comparing the effect of feature elimination on the model’s performance. Furthermore, we have specifically included All Features (gender, age, height, weight, BMI, NAH, ENA, PAH, EAH, d1, d2, and d3) to demonstrate the effectiveness of the genetic algorithm. Results from this ablation study are delineated in Table 3. The analysis reveals that NAH and ENA considerably influence model performance, whereas the impact of PAH and EAH is less significant. Even when PAH and EAH are excluded, the combination of NAH and ENA still demonstrates improved model performance.

Intriguingly, when all features were incorporated as input, the model’s accuracy was limited to 76.4%. A 6% improvement in model accuracy was observed following feature selection, underscoring the efficacy of utilizing genetic algorithms for this purpose.

**Table 3:** Results of ablation study

Features	Accuracy (%)	Sensitivity (%)	Specificity (%)	PPV (%)	NPV (%)
EAH	55.8	54.4	58.1	68.3	43.4
PAH	61.2	53.4	74.2	77.5	48.9
NAH	71.5	72.8	69.4	79.8	60.6
ENA	75.8	72.8	80.6	86.2	64.1
PAH + EAH	70.9	63.1	83.9	86.7	57.8
NAH + PAH	73.9	71.8	77.4	84.1	62.3
ENA + PAH	77.0	74.8	80.6	86.5	65.8
NAH + ENA	77.0	74.8	80.6	86.5	65.8
NAH + EAH	79.4	78.6	80.6	87.1	69.4
ENA + EAH	80.0	78.6	82.3	88.0	69.9
NAH + PAH + EAH	78.2	77.7	79.0	86.0	68.1
NAH + ENA + EAH	78.8	76.7	82.3	87.8	68.0
ENA + PAH + EAH	78.8	74.8	85.5	89.5	67.1
NAH + ENA + PAH	80.0	79.6	80.6	87.2	70.4
All Features	76.4	72.8	82.3	87.2	64.6
NAH + ENA + PAH + EAH	82.4	80.6	85.5	90.2	72.6

*PPV* denotes positive predictive value, *NPV* denotes negative predictive value, *NAH* Nasal-Acromion-Horizontal Angle, *ENA* Ear-Nasal-Acromion Angle, *PAH* Pupil-Acromion-Horizontal Angle, *EAH* Ear-Acromion-Horizontal Angle

## Discussion

To our knowledge, the evaluation model presented in this study is the inaugural model capable of distinguishing between adolescents with and without FHP. Our results demonstrate that the machine learning model, designed for the automated detection of FHP, demonstrates high predictive accuracy when applied to an independent adolescent sample from China. Moreover, the findings suggest that directly employing a deep learning model with the original image as input to predict FHP for adolescents is less efficacious than utilizing a machine learning model trained on features extracted from human body landmarks as input.

The amalgamation of computer vision, as embodied by Openpose, with the computational robustness of genetic algorithms, paves the way for a sophisticated, high-precision posture detection modality. The distribution of distinct angular features among FHP and non-FHP subjects, as illustrated in Fig. 3, emphasizes that the features extracted correspond accurately to the head and neck characteristics of FHP patients. This correspondence lends credence to the identified features’ validity. The deployment of this approach offers compelling advantages over existing methodologies, most notably by enhancing detection accuracy while obviating the need for physical contact or intrusive measures. As such, our findings not only reify the effectiveness of a OpenPose-based approach but also underscore its superiority in performance relative to conventional posture detection methods.

Decision tree and tree-based ensemble learning methods like random forest classifier have been widely used in disease prediction like cardiovascular disease [28], chronic kidney disease [29], heart-disease [30], coronary artery disease [31], and shows great performance, but in our study it didn’t outperform the extra tree classifier. In order to obtain results faster, we kept only the top three models with better performance in the phase of model selection. Random forests were eliminated, so it does not appear in the results. Reasons why the extra tree classifier is better than random forest is that in principle, it have more randomness than regular random forests. The extra tree classifier randomly selects a subset of features at each segmentation and then goes for the optimal branching attributes and branching thresholds, which outperforms random forests with a fixed number of feature subsets. This increase in randomness helps to create more decision trees that are independent of each other, thus improving the model performance. In addition, the extra tree classifier has better generalization properties and therefore gets better results on the test data.

Overall, the amalgamation of genetic algorithm and OpenPose algorithm unveils a promising path toward an innovative, non-contact approach for assessing head forward posture, which in turn has the potential to revolutionize the existing models of human posture evaluation and intervention. Our outcomes, though conclusive, invite future investigations to delve into further potentials of non-contact mechanisms, thereby advancing the field of human posture assessment and its associated health implications. The implications of our study extend to the fields of ergonomics, physical therapy, and computer-aided diagnostics, providing an innovative pathway for the detection and mitigation of posture-related conditions. As we move forward, we encourage future researchers to build upon this foundation, refining the key point detection-based detection approach and exploring its utility in a broader range of applications.

While the methodology proposed in our study has delivered promising results, it is imperative to acknowledge certain limitations that accompany these findings. Initially, the population under study was comprised of adolescent individuals, with the customary age bracket for this demographic being between 10 and 19 years. However, due to practical constraints related to school schedules and the availability of resources, the actual age spectrum of the participants was limited to those between 11 and 15 years old. Further, we adopted OpenPose for the extraction of body landmarks from images, yet the work of identifying the relevant features for FHP detection demands more extensive research. The current study marks a preliminary step in this direction, and subsequent investigations must delve deeper into feature selection for improved FHP detection. Lastly, OpenPose operates as a two-dimensional human pose estimation algorithm, which may inadvertently result in the loss of some spatial features pertinent to FHP. As we progress with our research, we intend to refine our methodology by employing a three-dimensional human pose estimation algorithm.

## Conclusion

In the culmination of our investigations, predicated upon the utilization of key reference points furnished by the Openpose platform, we have ascertained that a technique grounded in genetic algorithms demonstrates superior efficacy for non-contact diagnosis of a FHP. This research illuminates the potential for the integration of genetic algorithms in evolving the landscape of non-contact posture detection techniques, underscoring the magnitude of their applicability in this particular area.

## Data Availability

The datasets generated and/or analysed during the current study are not publicly available in order to preserve the privacy of the respondents but are available from the corresponding author on reasonable request.
